# Editorial: Oscillotherapeutics - toward real-time control of pathological oscillations in the brain

**DOI:** 10.3389/fnbeh.2022.1021616

**Published:** 2022-09-08

**Authors:** Yuichi Takeuchi, Qun Li, Takeshi Kawano, Jun Nagai, Tatsuya Mima

**Affiliations:** ^1^Department of Biopharmaceutical Sciences and Pharmacy, Faculty of Pharmaceutical Sciences, Hokkaido University, Sapporo, Japan; ^2^MTA-SZTE “Momentum” Oscillatory Neuronal Networks Research Group, Department of Physiology, University of Szeged, Szeged, Hungary; ^3^Department of Electrical and Electronic Information Engineering, Toyohashi University of Technology, Aichi, Japan; ^4^Laboratory for Glia-Neuron Circuit Dynamics, RIKEN Center for Brain Science, Saitama, Japan; ^5^The Graduate School of Core Ethics and Frontier Sciences, Ritsumeikan University, Kyoto, Japan

**Keywords:** oscillation, oscillopathy, neurological disorders, psychiatric disorders, closed-loop control, brain stimulation

**The aim of this Research Topic:** Oscillatory brain activities reflect and affect network activities in the brain. They support many physiological functions from motor control to cognition and emotion (Buzsáki, 2006; Nambu et al., 2020). Abnormal oscillatory brain activities are commonly observed in neurological and psychiatric disorders including epilepsy, Parkinson's disease, Alzheimer's disease, schizophrenia, anxiety/trauma-related disorders, major depressive disorders, and addiction (Leuchter et al., 2015; Takeuchi et al., 2021b). Therefore, these disorders can be considered as common oscillation defects “oscillopathies,” despite having distinct behavioral manifestations (Takeuchi and Berényi, 2020; Földi et al., 2021).

Recent advances in brain activity measurements and analyses have allowed us to study the pathological oscillations of each disorder as a possible biomarker of symptoms (Hultman et al., 2018). Furthermore, emerging brain stimulation technologies enable time- and space-targeted interventions of the pathological oscillations of neurological and psychiatric disorders. These interventions are possible therapeutic targets for regulating disorder symptoms (Tyler et al., 2018; Vöröslakos et al., 2018; Takeuchi et al., 2021a).

This Research Topic was organized to provide a comprehensive overview of pathological oscillations in neurological and psychiatric disorders. It was also aimed at examining correlations or causal relationships between pathological oscillations and the symptoms of disorders. These relationships were analyzed for the possible use of oscillations as biomarkers or therapeutic targets.

This Research Topic covers animal, human, and computational studies in the oscillotherapeutics field. Good animal models that accurately reflect the neurological and psychiatric symptoms of patients are necessary for providing the oscillotherapeutics proof-of-concept for future translational research. Clinical studies may test the proof-of-concept provided from animal research *via* solving clinical issues. Computational studies can prospectively or retrospectively provide a theory that explains pathological oscillations or their interventions.

**Statistics on this Research Topic:** The Research Topic was open from November 20, 2020 to December 31, 2021. It had been viewed over 37 thousand times at the time this editorial was prepared in June 2022. Ten articles were accepted after rigorous and constructive reviewing processes.

## Section 1: Reviews

Mokhothu and Tanaka gave a concise overview of physiological and non-physiological high frequency oscillations in hippocampus-related temporal lobe epilepsy in animals and humans.

Okonogi and Sasaki reviewed how neuronal oscillations in the limbic brain structure were engaged in emotional behaviors and altered by psychiatric changes such as anxiety and depression.

## Section 2: Animal studies

Acerbo et al. introduced a novel non-thermal electroporation technique to ablate neurons in a seizure focus with reduced vascular damage and accelerated tissue recovery.

Tomar et al. investigated the impacts of acute and chronic stress on spatial coding and gamma oscillations in the hippocampal CA1 region of mice.

Adlan et al. investigated sleep architecture and brain oscillatory patterns in a multiple-hit model for schizophrenic rats with impaired cognitive functions.

Wada et al. reported diminished “rubber tail task”-related expression of the immediate early gene (c-Fos) in the posterior parietal cortex of a transgenic mouse line that exhibits autistic-like phenotypes.

## Section 3: Clinical and computational studies

Nakajima et al. reported the case of a patient with Parkinson's disease whose local field potential fluctuations were less evident inside the hospital than outside after receiving chronic-adaptive deep brain stimulation of the subthalamic nuclei.

Ogawa et al. reported topographical distributions of desynchronization of electroencephalography recordings during volitional swallow in two amyotrophic lateral sclerosis patients.

Cimenser et al. reported that rhythmic gamma sensory stimulation improved the sleep and daily living activity of mild to moderate Alzheimer's disease patients.

Nobukawa et al. showed that applying chaotic resonance induced by a reduced region of orbit feedback signals may provide a promising treatment option for attention-deficit hyperactivity disorder.

## Summary

This is the first collection of articles in oscillotherapeutics research.

## Author contributions

All authors listed have made a substantial, direct, and intellectual contribution to the work and approved it for publication.

## Conflict of interest

The authors declare that the research was conducted in the absence of any commercial or financial relationships that could be construed as a potential conflict of interest.

## Publisher's note

All claims expressed in this article are solely those of the authors and do not necessarily represent those of their affiliated organizations, or those of the publisher, the editors and the reviewers. Any product that may be evaluated in this article, or claim that may be made by its manufacturer, is not guaranteed or endorsed by the publisher.
